# Supplementary material for: Content-rich biological network constructed by mining PubMed abstracts
Source: BMC Bioinformatics. 2004 Oct 8;5:147. doi: 10.1186/1471-2105-5-147 (PMC528731; doi:10.1186/1471-2105-5-147)
Supplement: Additional File 5 — The original Chilibot query results of the term "long-term potentiation (LTP)" and 22 other terms, limiting the latest references analyzed to the years 1990, 1995, 2000, and 2004. [file 1471-2105-5-147-S5.bz2 › chilibotAdditionalFile5/ltp2004/html/PLC_PKA.html]

 


 **PLC** and **PKA** 
  
Found 108 abstracts in PubMed,  **30 abstracts were retrieved and analyzed**.  


---

 Search Google  |
 PDF files only 
|  EDU domain only 

---

**Interactive relationship** (e.g. stimulation, inhibition, etc)

- In addition, exposure to pertussis toxin or exogenous activation of protein kinase A  **PKA**  inhibited  **PLC**  gamma1 tyrosine phosphorylation in response to Ca e.  Ref: 11934646 Bone, 2002
- Separate activation of the cAMP  **PKA**  pathway by 8 Br cAMP was completely prevented by PD098059 whereas activation of the  **PLC**  PKC pathway by the PKC activator dioctanoyl sn glycerol and the PKG pathway by 8 Br cGMP induced internalization of NaPi IIa which could be only partly blocked by PD 098059.  Ref: 12690463 Pflugers Arch, 2003
- We conclude that  **PLC**  and Na K ATPase are effector proteins for  **PKA**  and PKC, respectively, after stimulation of D 1 like receptors coupled to G s alpha proteins, in a sequence of events that begins with adenylyl cyclase  **PKA**  system activation followed by  **PLC**  PKC system activation.  Ref: 11997325 Am J Physiol Renal Physiol, 2002

- :-)

  **Parallel relationship** (e.g. studied together, co-existance, homology, etc.)

  - Conversely, the secretory responses of both ZG and AM preparations to VPAC2 ago were annulled by  **PLC**  I, lowered by PKC I, and unaffected by either AC I or  **PKA**  I.  Ref: 11836629 Int J Mol Med, 2002
  - The roles of specific downstream signals generated by the activated PTH PTH related protein PTHrP receptor PTH1R, such as cyclic adenosine monophosphate protein kinase A cAMP  **PKA**  and phospholipase C protein kinase C  **PLC**  PKC, in controlling RANKL and OPG expression and osteoclastogenesis remain uncertain.  Ref: 12211438 J Bone Miner Res, 2002
  - The current study investigated the effects of inhibiting adenylyl cyclase AC or cAMP dependent protein kinase  **PKA**  activities on the ability of NK cells to lyse K562 tumor cells, and on the activation of NK cell phospholipase C  **PLC**  by tumor targets.  Ref: 12941541 Hum Immunol, 2003
  - Treatment of NK cells with the  **PKA**  inhibitor, H 89, caused essentially complete inhibition of cytotoxic function while decreasing target induced stimulation of  **PLC**  by about 50%.  Ref: 12941541 Hum Immunol, 2003
  - Dopamine induced synaptic depression in the parabrachial nucleus is independent of CTX and PTX sensitive G proteins,  **PKA**  and  **PLC**  signalling pathways.  Ref: 14672813 Brain Res, 2004
  - Both  **PLC**  and  **PKA**  mediated pathways appear to regulate synaptic levels of DUNC 13 through controlling the rate of proteasome dependent DUNC 13 degradation.  Ref: 12532395 J Neurobiol, 2003
  - Inhibitors of phosphatidylinositide specific  **PLC**  U73122, guanylylcyclase ODQ,  **PKA**  Rp cAMPS or PLD 1 butanol did not affect the action of ATP.  Ref: 12956724 Eur J Neurosci, 2003
  - b  **PKA**  is a negative regulator of Ca e promoted  **PLC**  gamma1 phosphorylation.  Ref: 11934646 Bone, 2002
  - Role of cAMP  **PKA**   **PLC**  signaling cascade on dopamine induced PKC mediated inhibition of renal Na K ATPase activity.  Ref: 11997325 Am J Physiol Renal Physiol, 2002
  - At least two signalling cascades can be activated by PTH the  **PLC**  PKC and the cAMP  **PKA**  pathways.  Ref: 12690463 Pflugers Arch, 2003
  - To elucidate the second messenger pathway responsible for mediating the phase resetting actions of NT, we utilized the phospholipase C  **PLC**  and protein kinase A  **PKA**  inhibitors U 73122 and KT5720, respectively.  Ref: 12169114 Eur J Neurosci, 2002
